# Editorial: Mycobacterial Glycolipids—Role in Immunomodulation and Targets for Vaccine Development

**DOI:** 10.3389/fimmu.2020.603900

**Published:** 2020-10-08

**Authors:** Gunilla Källenius, Jérôme Nigou, Andrea Cooper, Margarida Correia-Neves

**Affiliations:** ^1^ Division of Infectious Diseases, Department of Medicine Solna, Karolinska Institutet, Stockholm, Sweden; ^2^ Institut de Pharmacologie et de Biologie Structurale, Université de Toulouse, Centre national de la recherche scientifique (CNRS), Université Paul Sabatier, Toulouse, France; ^3^ Leicester Tuberculosis Research Group (LTBRG), Department of Respiratory Sciences, University of Leicester, Leicester, United Kingdom; ^4^ Life and Health Sciences Research Institute (ICVS), School of Medicine, University of Minho, Braga, Portugal; ^5^ ICVS/Biomaterials, Biodegradables and Biomimetics (3B’s), Portugal (PT) Government Associate Laboratory, Braga, Portugal

**Keywords:** *Mycobacterium tuberculosis*, *Mycobacterium leprae*, glycolipid, lipoarabinomannan, Mycobacterium ulcerans, immunomodulation, vaccine

Diseases caused by mycobacteria, including tuberculosis (TB), leprosy, and Buruli ulcer, rank among the top causes of death and disability worldwide. One fourth of the world’s population is estimated to be infected with *Mycobacterium tuberculosis* (Mtb), leading to 1.4 million deaths annually due to TB (1).

Interrupting transmission of mycobacterial infections will reduce disease but requires prevention through successful vaccination, early detection of infected individuals, and the use of rapid and effective treatment (2). As rapid, low tech and definitive diagnostic tools are still lacking for all mycobacterial infections and the only vaccine currently in use is BCG, there is a continued need to maintain an intense research effort toward the design of novel and more effective vaccination strategies and diagnostic tools (3). While most of these efforts have focused on protein/peptide antigens (4), targeting unique carbohydrates and glycolipid antigens present in mycobacteria has emerged in recent years as a promising alternative strategy (5–7). In fact, some of the most successful vaccines against bacterial infections are based on carbohydrate antigens (8).

Glycolipids constitute a unique and important part of the mycobacterial cell envelope (9), and are some of the most potent mycobacterial immunomodulatory molecules (10). For instance, lipoarabinomannan (LAM) and its biosynthetic relatives, phosphatidylinositol mannosides (PIMs) and lipomannan (LM), exhibit important and unique immunomodulatory properties as agonists of several pattern recognition receptors (TLR2, DC-SIGN, Dectin-2) or as antigens of CD1-restricted T cells (11, 12). Trehalose dimycolate (TDM) is a highly immunostimulatory molecule and a potent adjuvant through its interaction with the C-type lectin mincle (13). In contrast, phenolic glycolipids (PGL) of several mycobacterial species down modulate the host innate immune response (14).

In this Research Topic, we have collected reviews and research reports from some of the key investigators who are increasing our knowledge on the role of mycobacterial glycolipids and how they influence the immune response and modulate both immunity and pathogenesis related with the important mycobacterial diseases.

Garcia-Vilanova et al. provide us with an excellent overall view of how the unique glycolipids of Mtb impact virulence, pathogenesis, and drug resistance. They also develop an exciting working model of how these glycolipids may dictate the evolution of the Mtb cell envelope from ancient to modern strains. In a particularly insightful manner, this review also addresses how these Mtb cell envelope glycolipids are altered in response to the environment that the bacteria finds itself in when first entering the human lung. Key observations include the potential for the host lung alveolar environment to alter the glycolipids and for the role of these molecules in adjusting the hydrophobicity of the Mtb in response to the lung environment. Finally, the authors highlight the role of these molecules in driving key early immune responses in the lung and thereby promoting successful infection.

Correia-Neves et al. bring to the fore the specific role of the dominant glycolipid LAM in development of TB. This molecule has been the target of investigations for a long time and we are now beginning to understand the ability of this molecule to promote and manipulate immune responses. Of particular interest in the case of the mannose-capped arabinan moiety of LAM is their role in the development of novel vaccine candidates against TB. The attractive aspects of LAM and its structural variants in this regard lie in its recently described capacity to activate human CD1b-restricted T cells and B cells with anti-LAM antibodies being induced during both Mtb infection and after BCG vaccination. This capacity to induce both non-polymorphic and humoral responses, and the ability of monoclonal antibodies against LAM to confer protection in animal models, support the need for continued development of this agent in the drive to develop vaccines that utilize the full range of the immune response to combat this disease.

*Mycobacterium leprae*, the causative agent of leprosy, is unique among human pathogens in its capacity to produce the virulence factor glycolipid PGL-I. In addition to mediating the unique tropism of this bacteria for neurons, PGL-I interacts with complement receptor (CR)3 on macrophages to promote infection. In an exciting and impactful research report, Doz-Deblauwe et al. show that PGL-I binding to CR3 also enhances bacterial invasion of both polymorphonuclear neutrophils and dendritic cells. They identify the CR3-Syk-NFATc axis as a novel signaling pathway activated by PGL-I in innate immune cells and show that this fundamentally rewires the host cytokine responses to *M. leprae*. By defining the mechanism by which glycolipids manipulate the human immune response, we are gaining not only useful information in the fight against mycobacterial disease but also defining the pathways by which our body balances the need to eliminate the bacteria while maintaining tissue integrity.

Classical models of mycobacterial immunity stress the need for T cells that respond to mycobacterial peptide antigens and exhibit classical features of cellular immune responses; we have a good understanding of this response in humans and animal models. Recently, T cells that recognize lipid and metabolite antigens and that exhibit more innate characteristics, are under investigation and our understanding of their role in the immune response to TB is being refined. Specifically, T cells that recognize mycobacterial glycolipid antigens (mycolipids) can confer protection following Mtb infection in animal models; these T cells share biological characteristics with both adaptive and innate-like T cells. In a thorough and compelling review, James and Seshadri collate the existing evidence supporting the concept that mycolipid-specific T cells exist on a spectrum of “innateness”. Defining this spectrum and determining the capacity of these cells will allow the field to develop rational strategies to leverage these novel cells into new diagnostics and vaccines for mycobacterial diseases.

As highlighted here, the mycobacteria can use their unique carbohydrate and glycolipid molecules to manipulate the immune response to allow persistence without inducing too much tissue damage (15). Mtb is particularly focused on this and we need to understand how best to induce a protective long-lasting immune response without inducing overt lung inflammation. The presence of inducible bronchus-associated lymphoid tissue (iBALT) correlates with protection from Mtb infection. Here, in an exciting and thought provoking research article using non-human primates infected with a transposon mutant library of Mtb, Dunlap et al. determined that the mutants present within the iBALT-containing granulomas were those associated with alterations in cell wall lipid transport and metabolism. Following up on this observation, the authors determined that an Mtb glycolipid factor participates in the induction of iBALT formation during TB, by directly modulating cytokine and chemokine production in host cells.

Finally, Mtb envelope components are not only encountered at the Mtb-host direct contact, but are also released from the bacterial envelope and can be detected in the endosomal compartments of the infected cells or in extracellular vesicles produced by Mtb itself or by infected cells. In a comprehensive review of this emerging and important area, Layre presents the current knowledge on Mtb envelope components with attention to how these molecules are shuttled within extracellular vesicle; how these vesicles impact host immune cells and where the gaps are in our understanding. This review brings us full circle by describing how the unique mycobacterial glycolipid molecules that are capable of responding to the environment, manipulating the immune response and may provide novel avenues for diagnostic and vaccine development are delivered not only at the interface between bacterium and host but throughout the body.

## Author Contributions

All authors contributed to the article and approved the submitted version.

## Conflict of Interest

The authors declare that the research was conducted in the absence of any commercial or financial relationships that could be construed as a potential conflict of interest.

## References

[1] WHO. World Health Organization Global Tuberculosis Report 2019. (World Health Organization) (2019). Available at: https://www.who.int/tb/publications/global_report/tb19_Exec_Sum_12Nov2019.pdf?ua=1.

[2] WHO. World Health Organization Global strategy and targets for tuberculosis prevention, care and control after 2015. (World Health Organization) (2014). Available at: https://www.who.int/tb/post_2015_tb_presentation.pdf.

[3] Lienhardt C Lonnroth K Menzies D Balasegaram M Chakaya J Cobelens F Translational Research for Tuberculosis Elimination: Priorities, Challenges, and Actions. PloS Med (2016) 13:e1001965. 10.1371/journal.pmed.1001965 26933883 PMC4775029

[4] Andersen P Scriba TJ Moving tuberculosis vaccines from theory to practice. Nat Rev Immunol (2019) 19:550–62. 10.1038/s41577-019-0174-z 31114037

[5] Correia-Neves M Froberg G Korshun L Viegas S Vaz P Ramanlal N Biomarkers for tuberculosis: the case for lipoarabinomannan. ERJ Open Res (2019) 5:00115–2018. 10.1183/23120541.00115-2018 PMC6368998 30775376

[6] Layre E Mazurek J Gilleron M Glycolipid Presentation by CD1. In: eLS. Chichester: John Wiley & Sons, Ltd (2015).

[7] Moody DB Suliman S CD1: From Molecules to Diseases. F1000Research (2017) 6:1909. 10.12688/f1000research.12178.1 29152228 PMC5664979

[8] Berti F Micoli F Improving efficacy of glycoconjugate vaccines: from chemical conjugates to next generation constructs. Curr Opin Immunol (2020) 65:42–9. 10.1016/j.coi.2020.03.015 32361591

[9] Minnikin DE Brennan PJ Lipids of Clinically Significant Mycobacteria. In: Goldfine H, editor. Health Consequences of Microbial Interactions with Hydrocarbons, Oils, and Lipids. Cham: Springer Nature Switzerland AG (2020). p. 1–76.

[10] Kasmar AG Layre E Moody DB Lipid adjuvants and antigens embedded in the mycobacterial cell envelope. In: Norazmi MN Acosta A Sarmiento ME, editors. The Art & Science of Tuberculosis Vaccine Development 2nd Edition. Oxford: Oxford University Press (2014). p. 123–49.

[11] Vergne I Gilleron M Nigou J Manipulation of the endocytic pathway and phagocyte functions by Mycobacterium tuberculosis lipoarabinomannan. Front Cell Infect Microbiol (2015) 4:187. 10.3389/fcimb.2014.00187 25629008 PMC4290680

[12] Gilleron M Jackson M Nigou J Puzo G Structure, Biosynthesis, and Activities of the Phosphatidyl-myo-Inositol-Based Lipoglycans. In: Daffé M Reyrat JM, editors. The mycobacterial cell envelope. Washington: ASM Press (2008). p. 75–105.

[13] Ishikawa E Mori D Yamasaki S Recognition of Mycobacterial Lipids by Immune Receptors. Trends Immunol (2017) 38:66–76. 10.1016/j.it.2016.10.009 27889398

[14] Oldenburg R Demangel C Pathogenic and immunosuppressive properties of mycobacterial phenolic glycolipids. Biochimie (2017) 141:3–8. 10.1016/j.biochi.2017.03.012 28322927

[15] Orme IM Robinson RT Cooper AM The balance between protective and pathogenic immune responses in the TB-infected lung. Nat Immunol (2015) 16:57–63. 10.1038/ni.3048 25521685

